# Effects of Positive End-Expiratory Pressure on Lung Recruitment, Respiratory Mechanics, and Intracranial Pressure in Mechanically Ventilated Brain-Injured Patients

**DOI:** 10.3389/fphys.2021.711273

**Published:** 2021-10-18

**Authors:** Chiara Robba, Lorenzo Ball, Stefano Nogas, Denise Battaglini, Antonio Messina, Iole Brunetti, Giuseppe Minetti, Lucio Castellan, Patricia R. M. Rocco, Paolo Pelosi

**Affiliations:** ^1^Anesthesia and Intensive Care, San Martino Policlinico Hospital, Istituto di Ricovero e Cura a Carattere Scientifico (IRCCS) for Oncology and Neurosciences, Genoa, Italy; ^2^Department of Surgical Sciences and Integrated Diagnostics (DISC), University of Genoa, Genoa, Italy; ^3^Humanitas Clinical and Research Center—Istituto di Ricovero e Cura a Carattere Scientifico (IRCCS), Rozzano, Italy; ^4^Radiology Department San Martino Policlinico Hospital, Istituto di Ricovero e Cura a Carattere Scientifico (IRCCS) for Oncology and Neurosciences, Genoa, Italy; ^5^Laboratory of Pulmonary Investigation, Carlos Chagas Filho Institute of Biophysics, Federal University of Rio de Janeiro, Rio de Janeiro, Brazil

**Keywords:** positive end expiratory pressure, intracranial pressure, brain injured patients, quantitative computed tomography, mechanical ventilation

## Abstract

**Background:** The pathophysiological effects of positive end-expiratory pressure (PEEP) on respiratory mechanics, lung recruitment, and intracranial pressure (ICP) in acute brain-injured patients have not been completely elucidated. The primary aim of this study was to assess the effects of PEEP augmentation on respiratory mechanics, quantitative computed lung tomography (qCT) findings, and its relationship with ICP modifications. Secondary aims included the assessment of the correlations between different factors (respiratory mechanics and qCT features) with the changes of ICP and how these factors at baseline may predict ICP response after greater PEEP levels.

**Methods:** A prospective, observational study included mechanically ventilated patients with acute brain injury requiring invasive ICP and who underwent two-PEEP levels lung CT scan. Respiratory system compliance (Crs), arterial partial pressure of carbon dioxide (PaCO_2_), mean arterial pressure (MAP), data from qCT and ICP were obtained at PEEP 5 and 15 cmH_2_O.

**Results:** Sixteen examinations (double PEEP lung CT and neuromonitoring) in 15 patients were analyzed. The median age of the patients was 54 years (interquartile range, IQR = 39–65) and 53% were men. The median Glasgow Coma Scale (GCS) at intensive care unit (ICU) admission was 8 (IQR = 3–12). Median alveolar recruitment was 2.5% of total lung weight (−1.5 to 4.7). PEEP from 5 to 15 cmH_2_O increased ICP [median values from 14.0 (11.2–17.5) to 23.5 (19.5–26.8) mmHg, *p* < 0.001, respectively]. The amount of recruited lung tissue on CT was inversely correlated with the change (Δ) in ICP (rho = −0.78; *p* = 0.0006). Additionally, ΔCrs (rho = −0.77, *p* = 0.008), ΔPaCO_2_ (rho = 0.81, *p* = 0.0003), and ΔMAP (rho = −0.64, *p* = 0.009) were correlated with ΔICP. Baseline Crs was not predictive of ICP response to PEEP.

**Conclusions:** The main factors associated with increased ICP after PEEP augmentation included reduced Crs, lower MAP and lung recruitment, and increased PaCO_2_, but none of these factors was able to predict, at baseline, ICP response to PEEP. To assess the potential benefits of increased PEEP in patients with acute brain injury, hemodynamic status, respiratory mechanics, and lung morphology should be taken into account.

## Introduction

A substantial number of patients with acute brain injury require mechanical ventilation (Borsellino et al., 2016) due to both neurological and respiratory causes (Della Torre et al., 2017). The aim of mechanical ventilation is to optimize oxygen delivery and minimize lung and brain injury (Frisvold et al., 2019). The use of lung-protective ventilation strategies has been shown to reduce morbidity and mortality in acute respiratory distress syndrome (ARDS) and non-ARDS critically ill patients (Sutherasan et al., 2014; Serpa Neto et al., 2015; Simonis et al., 2018). However, strategies comprising the use of high positive end-expiratory pressure (PEEP) have been challenged in brain-injured patients because of concerns regarding their effects on cerebral hemodynamics (Nemer et al., 2011; Borsellino et al., 2016; Robba et al., 2020), in particular intracranial pressure (ICP). Possible mechanisms responsible for ICP augmentation after PEEP application include alveolar overdistension with the increase of arterial partial pressure of carbon dioxide (PaCO_2_) levels, and hemodynamic instability (Caricato et al., 2005; Mascia et al., 2005; Nemer et al., 2011). To date, the pathophysiological interplay between intracranial changes, respiratory system mechanics, and alveolar recruitment has not been completely elucidated, and no specific recommendations are available regarding the optimal levels of PEEP to be applied in acute brain-injured patients (Robba et al., 2020). We, therefore, conducted an observational study whose primary aim was to investigate the effects of two levels of PEEP (5 and 15 cmH_2_O) on respiratory mechanics, quantitative lung computed tomography (qCT) findings, and its relationship with ICP changes in brain-injured patients. Secondary aims included the assessment of the correlation between different factors (respiratory mechanics and qCT features) with the changes of ICP, and how these factors at baseline may predict ICP response after greater PEEP levels. Finally, we explored whether non-invasive neuromonitoring tools are able to assess changes of ICP following augmented PEEP levels. We hypothesized that the effect of greater PEEP levels on ICP depends on the amount of alveolar recruitment and respiratory mechanics.

## Methods

We followed the “Strengthening the Reporting of Observational Studies in Epidemiology (STROBE)” statement guidelines for observational cohort studies (Supplementary Table 1) (von Elm et al., 2014). This study was performed at San Martino Policlinico Hospital, Genoa, Italy, a tertiary academic hospital with neurocritical care facilities, from August 1, 2020, to March 8, 2021. The study was approved by the ethics review board “Comitato Etico Regione Liguria” (protocol n. CER Liguria: 23/2020). According to the local regulations, written consent was obtained from next of kin of the patients, as all patients were unconscious at the time of inclusion.

### Inclusion and Exclusion Criteria

Inclusion criteria were critically ill adult patients who required intubation and invasive mechanical ventilation following acute brain injury (traumatic brain injury, TBI; subarachnoid hemorrhage, SAH; intracranial hemorrhage, ICH) admitted to the intensive care unit (ICU), requiring invasive ICP and other neuromonitoring tools (Transcranial Doppler, TCD and optic nerve sheath diameter, ONSD) and who underwent two-PEEP CT scan based on clinical indication with PEEP 5 and 15 cmH_2_O. Exclusion criteria were the absence of informed consent; the absence of indications for invasive ICP monitoring (i.e., coagulopathy); the absence of temporal window for TCD evaluation; basal skull fracture with the cerebrospinal fluid leak, or ocular trauma for ONSD measurement; patients requiring contrast medium during CT for clinical reasons or having contraindications to higher PEEP (e.g., emphysema and undrained pneumothorax), or judged too instable to be safely transported to the CT facility (e.g., hemodynamic instability, need for high doses vasopressors, or acute and refractory increased ICP).

### Data Collection and Patients' Management

Demographic, epidemiologic, and clinical data were obtained from electronic medical records of patients and collected by physicians trained in critical care at admission to the ICU and on the day when a double PEEP CT scan was obtained. Recorded data included admission demographics [age, gender, and body mass index (BMI)], comorbidities (asthma, chronic respiratory disease, hypertension, chronic cardiac disease diabetes mellitus, chronic kidney injury, and previous neurological disease), type of brain injury, neurological status at ICU admission (Glasgow Coma Scale, GCS), type of ICP monitoring (intraparenchymal and external ventricular drain), ICU complications, ICU length of stay (LOS), and Glasgow Outcome Score (GOS) at ICU discharge. Patients were sedated with propofol and/or midazolam and fentanyl, targeting the tidal volume of 6–8 ml per kg of predicted body weight (PBW), but increases were tolerated based on the driving pressure. The respiratory rate was titrated to maintain pH between 7.35 and 7.45. On the day of CT scan, ventilatory data, respiratory mechanics, and blood gases parameters (i.e., inspired fraction of oxygen (FiO_2_), PEEP, plateau pressure (Pplat), respiratory system compliance (Crs), tidal volume (VT), respiratory rate (RR), arterial and venous saturation of oxygen (SaO_2_ and SvO_2_), arterial pH (pHa), partial pressure of oxygen (PaO_2_ and PaCO_2_) were obtained at PEEP = 5 cmH_2_O (T0) and at PEEP = 15 cmH_2_O (T1). Vital signs, such as mean arterial pressure (MAP) and neuromonitoring parameters [ICP, ONSD, systolic, mean, and diastolic flow velocities (FVs, FVd, and FVm)] were also collected at T0 and T1.

### Clinical Rationale for PEEP Test

The decision to perform a PEEP test was based on the judgment of the treating physician, if optimization of mechanical ventilation was required. PEEP test was performed in Volume-Controlled Ventilation aiming to target the tidal volume of 6–8 ml per kg/PBW. To date, no universal recommendations are available concerning the optimal PEEP levels in the invasively ventilated brain-injured patients (Robba et al., 2020). Therefore, in our institution, a PEEP test is performed to increasing PEEP from 5 to 15 cmH_2_O, assessing both respiratory mechanics and cerebral hemodynamics. These values have previously been demonstrated to be safe and can lead to increased brain oxygenation, without the increase in ICP (Nemer et al., 2011). However, as greater PEEP levels may result in worsening of the respiratory mechanics with eventually increased alveolar hyperdistention (Mascia et al., 2005), two-PEEP CT, when possible, has become part of our routine clinical evaluation and has been performed in our institution in other groups of patients (Ball et al., 2021). Evaluation and calculation of gas exchanges, respiratory mechanics, and details on the protocol for two-PEEP CT acquisition and analysis are described in the ESM.

### CT Scan Acquisition and Analysis

Images were acquired during expiratory breath-hold at 5 and 15 cmH_2_O. The two scans were acquired in sequence, interleaved by 1–2 min of uninterrupted ventilation at PEEP 15 cmH_2_O (Ball et al., 2021). This time of ventilation at 15 cmH_2_O of PEEP was applied before repeating the CT scan (T1). For safety reasons, no recruitment maneuver was performed.

Lung segmentation was performed excluding big airway, vessels, and pleural effusion. Segmentations were performed using ITKSnap (http://www.itksnap.org), image analysis was performed with Matlab scripts (Mathworks, MA, USA), based on widely adopted numerical methods (Malbouisson et al., 2001). Alveolar recruitment was defined as the difference in the non-aerated compartment from PEEP 5–15 cmH_2_O, divided by total lung weight at PEEP of 5 cmH_2_O (Gattinoni et al., 2006).

### Neuromonitoring

The indications for invasive ICP placement followed the latest Brain Trauma Foundation Guidelines (Carney et al., 2016). Ultrasound measurement was performed by a selected group of experienced operators (CR, SN, and DB) using a standardized insonation technique to reduce inter-operator variability. Ultrasound measurements were performed after PEEP augmentation and after repeating the second CT.

Transcranial Doppler was performed bilaterally on the middle cerebral artery (MCA) through the temporal window using a traditional 2-MHz transducer (Philips SparQ®) as previously described (Robba et al., 2017b). Non-invasive ICP estimation using TCD (ICP_TCD_) was obtained using a previously validated formula (Rasulo et al., 2017). Ultrasound examination of the ONSD was performed using a 7.5 MHz linear ultrasound probe (Philips SparQ®) using the lowest possible acoustic power that could measure the ONSD. The probe was oriented perpendicularly in the vertical plane and at around 30° in the horizontal plane on the closed eyelids of both eyes of subjects in the supine position. Ultrasound gel was applied on the surface of each eyelid, and the measurements were made in the axial and sagittal planes of the widest diameter visible 3 mm behind the retina in both eyes. The final ONSD value was calculated as previously described (Robba et al., 2016, 2017a).

### Statistical Analysis

An *a priori* sample size calculation was not feasible due to the lack of data on quantitative CT analysis in brain-injured patients, but our sample size was similar to previous physiologic studies regarding PEEP augmentation in ARDS or brain-injured patients (Mascia et al., 2005; Nemer et al., 2011; Mauri et al., 2016, 2020). Data are reported as median (interquartile range, IQR), if not otherwise specified. We compared data between groups with the Mann–Whitney *U* or Fisher's exact test, as appropriate. Variables acquired at two-PEEP levels were compared with the Wilcoxon signed-rank test. Changes of variables from PEEP 5 to PEEP 15 were calculated as Δ (value at PEEP 15 cmH_2_O–value at PEEP 5 cmH_2_O). Correlations were sought using Spearman's rho. A linear regression analysis was performed using ΔICP (invasive ICP) as the dependent variable and alveolar recruitment, ΔMAP, Crs, and PaCO_2_ as independent variables. As an exploratory analysis, we modeled ΔICP as a function of clinically sound covariates using linear regression, adopting a variance inflation factor threshold of 5 as an acceptable limit for multi-collinearity. All statistical analyses were performed in SPSS Statistics, Version 25.0 (IBM Corp., Armonk, NY, USA). Significance was assumed at two-tailed *p* < 0.05.

## Results

### General Characteristics

A total of 16 examinations (double PEEP quantitative CT and neuromonitoring, including invasive ICP, ONSD, and TCD) from 15 patients were included in the analysis (in one patient, the measures were obtained twice). The median age of the patients was 54 years (IQR = 39–65); 53.3% were men. Six patients (40%) were admitted for SAH, six after TBI (40%), and three (20%) for ICH (Table 1). The median GCS was eight (IQR = 3–12); intraparenchymal and intraventricular monitoring were inserted in seven (46.6%) and eight (53.3%) cases, respectively. One patient died in ICU (6.6%) and the median ICU LOS was 16 (IQR = 13–21) days.

**Table 1:** Characteristics of the patients at ICU admission.

**Demographics**	
Gender, male, *n* (%)	8 (53.3%)
Age [years], median [IQR]	54 [39–65]
BMI [kg/m^2^], median [IQR]	26.3 [25.5–27.9]
PBW [kg], median [IQR]	68.7 [57–78]
**Comorbidities**	
Respiratory disease, *n* (%)	3 (20)
Cardiovascular disease, *n* (%)	1 (6.6)
Cancer, *n* (%)	0 (0)
Neurologic disorders, *n* (%)	1 (6.6)
Moderate/severe liver disease, *n* (%)	1 (6.6)
End-stage kidney injury, *n* (%)	0 (0)
Hypertension, *n* (%)	7 (46.6)
Diabetes mellitus, *n* (%)	3 (20)
**ICU characteristics**	
**Reason for ICU admission, *n* (%)**	
TBI	6 (40)
SAH	6 (40)
ICH	3 (20)
GCS score, median [IQR]	8 [3–12]
**Type of ICP monitor, *n* (%)**	
Bold	7 (46.6)
EVD	8 (53.3)
Need for vasopressors, *n* (%)	13 (86.7)
**ICU complications**	
Respiratory failure, *n* (%)	1 (6.6)
Ventilator- associated pneumonia, *n* (%)	4 (26.6)
Cardiovascular, *n* (%)	2 (13.3)
Acute kidney injury, *n* (%)	0 (0)
Sepsis, *n* (%)	1 (6.6)
Vasospasm, *n* (%)	2 (13.3)
**ICU discharge characteristics**	
Mortality, *n* (%)	1 (6.6)
GOS, median [IQR]	4 [3–4]
ICU length of stay, median [IQR]	16 [13–21]

*IQR, Interquartile range; n, number; BMI, body mass index; PBW, predicted body weight; ICU, intensive care unit; TBI, traumatic brain injury; SAH, subarachnoid hemorrhage; ICH, intracranial hemorrhage; GCS, Glasgow Coma Scale; ICP, intracranial pressure; EVD, external ventricular drain; GOS, Glasgow outcome score*.

### Effect of PEEP Augmentation on Respiratory Mechanics, Quantitative CT Findings, and ICP

**Figure 1:**
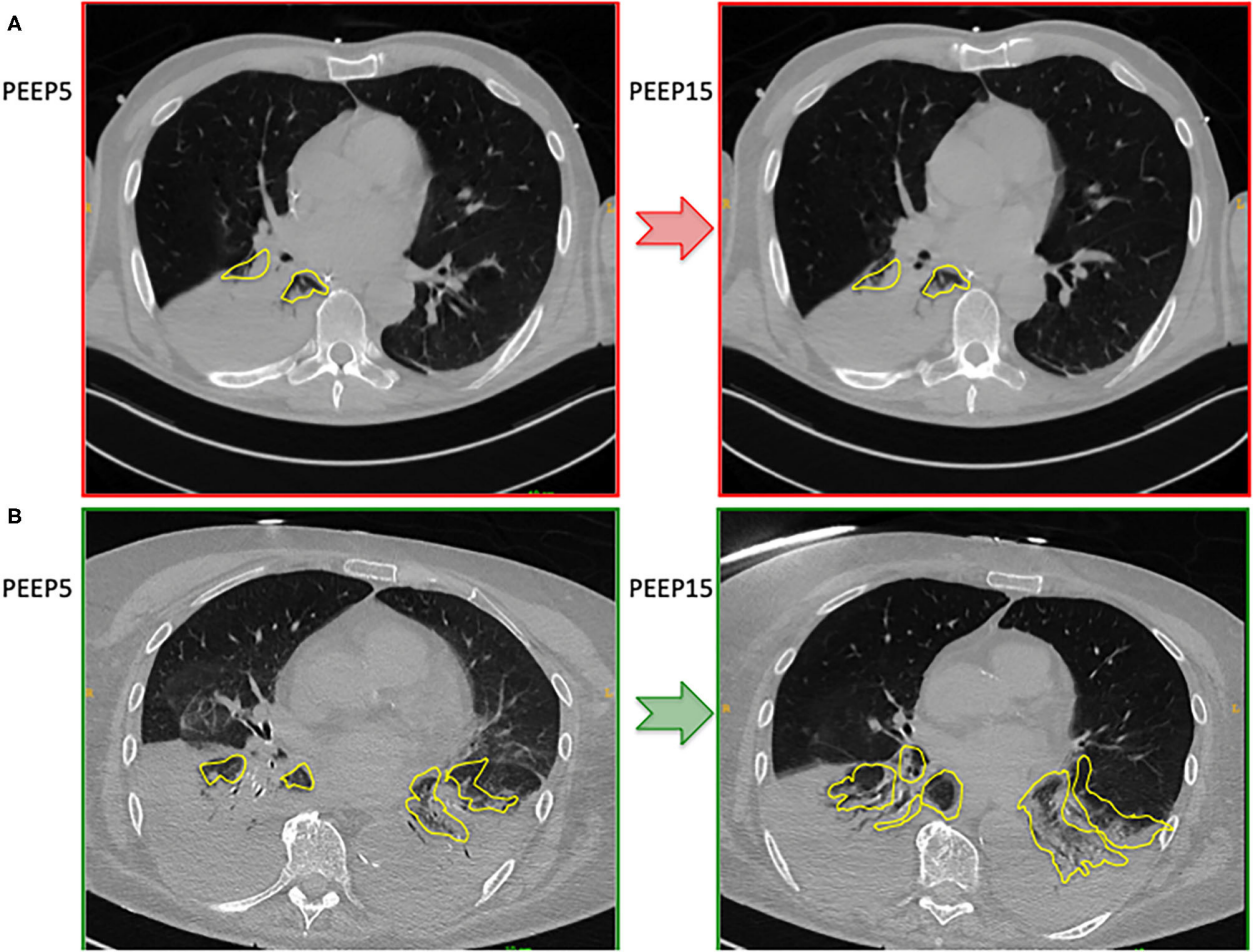
Representative cases representing CT images at 5 and 15 cmH_2_O of PEEP in a patient with poor alveolar recruitment **(A)** and good alveolar recruitment **(B)**.

**Figure 2:**
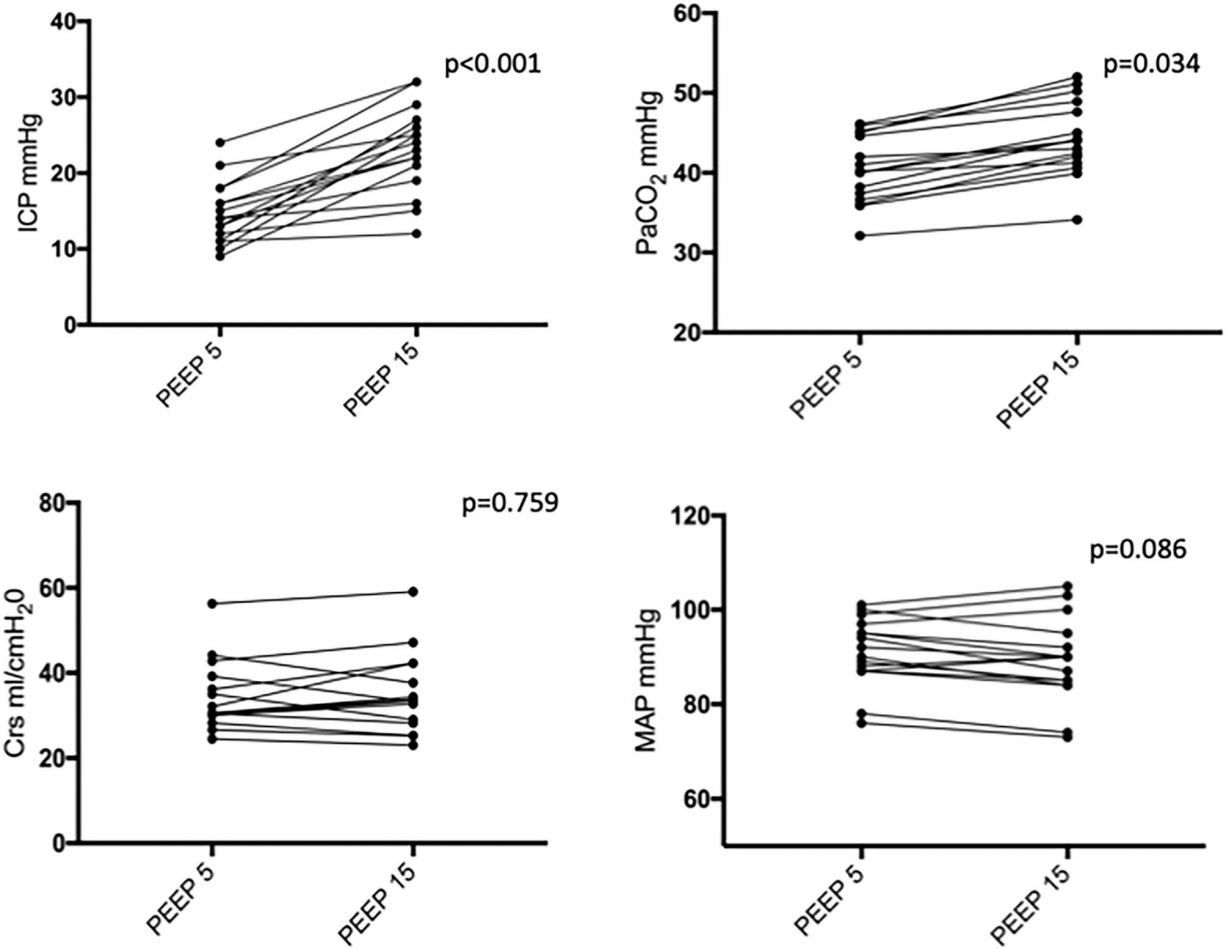
Intracranial pressure (ICP), partial pressure of carbon dioxide (PaCO_2_), respiratory system compliance (Crs), and mean arterial pressure (MAP) at PEEP of 5 and 15 cmH_2_O. Black dots and lines represent individual patient data. PEEP, positive end-expiratory pressure.

**Figure 3:**
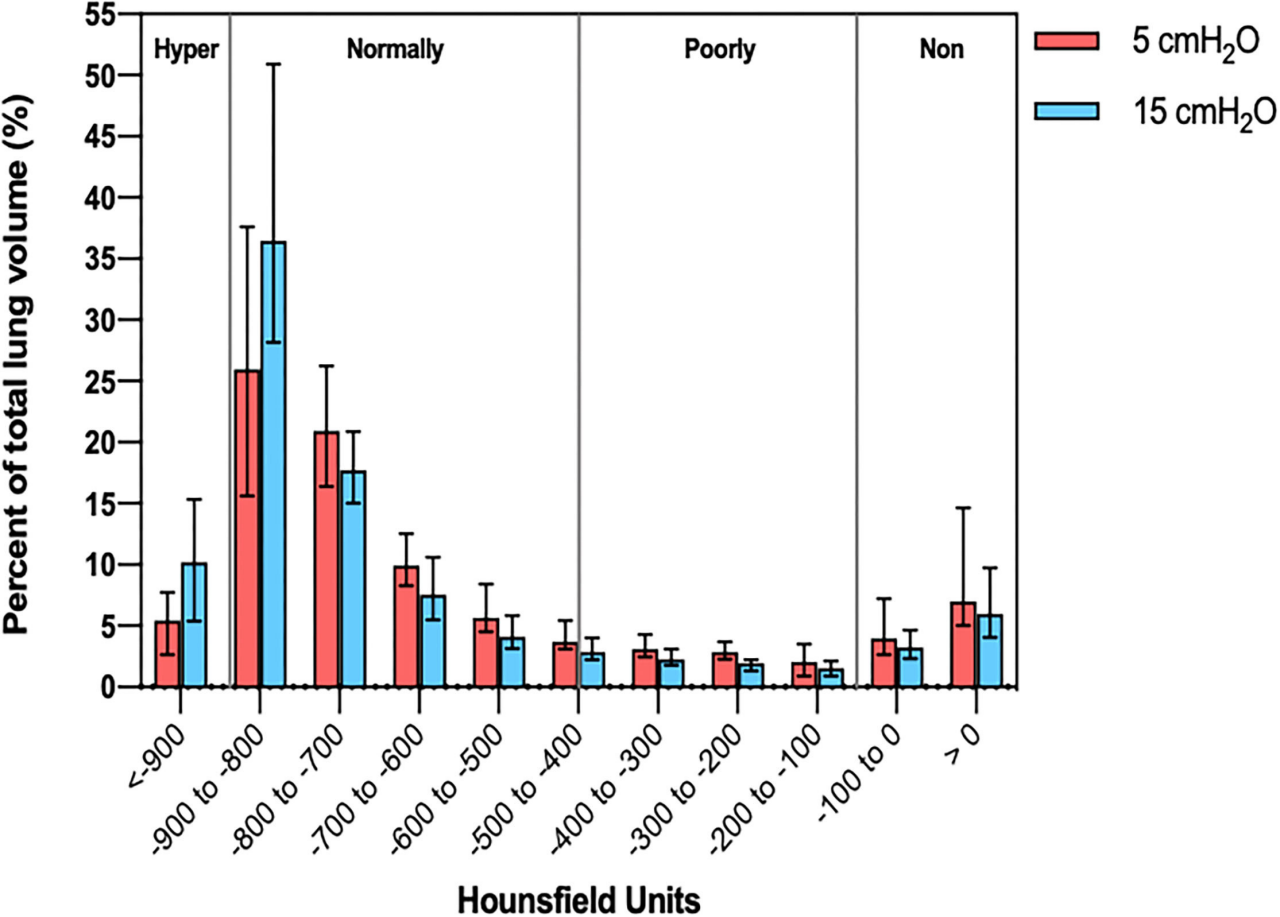
Histogram distribution of lung volume aeration at 5 and 15 cmH_2_O of PEEP. PEEP, positive end-expiratory pressure.

Figure 1 shows two representative examples of CT images at 5 and 15 cmH_2_O of PEEP in two patients with low and high alveolar recruitment. After greater PEEP levels, systemic oxygenation [PaO_2_, from 96.4 (81–108) to 98 (85.4–148) mmHg; *p* = 0.039], and PaCO_2_ [from 40 (36.8–44.9) to 44 (41.4–48.6) mmHg; *p* = 0.034] increased, while median Crs did not change (Table 2 and Figure 2). Total lung volume was augmented after greater PEEP levels, as also the gas volume, but not total lung weight (Table 2). Median alveolar recruitment was 2.5% (−1.5–4.7). Figure 3 illustrates the frequency distribution of Hounsfield units at 5 and 15 cmH_2_O of PEEP. The increase of PEEP from 5 to 15 cmH_2_O resulted in higher median invasive ICP values [14 (11.2–17.5) vs. 23.5 (19.5–26.8) mmHg, *p* < 0.001; Figure 2] and non-invasive ICP measured through TCD and of ONSD values. Higher PEEP also resulted in a significant reduction of cerebral perfusion pressure (CPP) [78 (71–81.7) vs. 63 (57.8–74.8) mmHg, *p* = 0.001; Table 2].

**Table 2:** Ventilator settings, respiratory mechanics, arterial blood gases, quantitative CT analysis, neuromonitoring data, and hemodynamics in our cohort at PEEP = 5 and 15 cmH_2_O.

**Parameter**	**PEEP = 5**	**PEEP = 15**	***p***
	**(*N* = 16)**	**(*N* = 16)**	
**Ventilator settings and arterial blood gases**			
Tidal volume, median [IQR], ml/kg PBW	7.4 [6.9–7.9]	7.4 [6.9–7.9]	0.999
Respiratory rate, median [IQR], 1/min	21 [18–23]	21 [18–22]	0.257
Plateau pressure, median [IQR], cmH_2_0	21 [18–22]	30 [28–34]	<0.001
Respiratory system compliance, median [IQR], ml/cmH_2_0	30 [30–38]	34 [28–41]	0.759
Venous admixture, median [IQR], (%)	25.7 [15.1–32.3]	21.6 [15–31.2]	0.717
Ventilation ratio, median [IQR]	1.7 [1.5–2]	1.9 [1.7–2.2]	0.109
pH, median [IQR]	7.41 [7.36–7.45]	7.45 [7.39–7.48]	0.343
PaO_2_, median [IQR], mmHg	96 [81–108]	98 [85–148]	0.039
SaO_2_, median [IQR], mmHg	98 [97–100]	99 [97–99]	0.724
PaCO_2_, median [IQR], mmHg	40 [37–45]	44 [41–49]	0.034
SvO_2_, median [IQR], mmHg	72 [63–78]	75 [68–78]	0.453
PaO_2_/FiO_2_, median [IQR], mmHg	195 [163–216]	195 [171–296]	0.049
**Quantitative computed tomography analysis**			
Total lung volume (ml)	2,704 [2,360–3,574]	3,334 [2,883–4,228]	0.001
Total lung weight (g)	1,076 [915–1,368]	1,010 [884–1,365]	0.679
Gas volume (ml)	1,693 [1,204–2,292]	2,429 [1,862–2,864]	<0.001
Mean attenuation (HU)	−601 [-671 –−557]	−677 [-724 –−618]	0.001
Hyper-aerated tissue (g)	10 [3–17]	23 [14–32]	0.002
Hyper-aerated tissue (% of total lung weight)	0.8 [0.3–1.5]	2 [1.4–2.3]	0.001
Normally aerated tissue (g)	449 [382–592]	533 [402–655]	0.002
Normally aerated tissue (% of total lung weight)	45 [31.4–54.2]	48 [35–55]	0.008
Poorly aerated tissue (g)	250 [193–307]	199 [167–278]	0.023
Poorly aerated tissue (% of total lung weight)	22.4 [16.6–26.1]	19.7 [13.1–21.6]	0.017
Non-aerated tissue (g)	434 [213–563]	344 [189–567]	0.121
Non-aerated tissue (% of total lung weight)	30.1 [25–44.6]	31.2 [21.2–39]	0.017
**Neuromonitoring**			
ICP, median [IQR], mmHg	14 [11–17]	23 [19–26]	<0.001
CPP, median [IQR], mmHg	78 [71–82]	63 [58–75]	0.001
FVs, median [IQR], cm/s	112 [106–121]	97 [55–116]	0.036
FVd, median [IQR], cm/s	43 [32–51]	19 [15–27]	0.001
FVm, median [IQR], cm/s	65 [59–74]	46 [31–56]	0.001
ONSD, median [IQR], mm	4.5 [4.1–5.1]	5.8 [5.4–6.4]	0.001
ICP_TCD_, median [IQR], mmHg	21 [18–25]	33 [31–45]	0.001
**Hemodynamics**			
Mean arterial pressure, median [IQR], mmHg	91 [87–97]	90 [84–94]	0.086

*Data are presented as median [IQR, interquartile range]. IQR, interquartile range; PaO_2_, arterial partial pressure of oxygen, SaO_2_, arterial oxygen saturation; PaCO_2_, arterial partial pressure of carbon dioxide; SvO_2_, venous saturation of oxygen; PaO_2_/FiO_2_ (inspired fraction of oxygen); HU: Hounsfield Units; ICP, intracranial pressure; FVs, FVd, FVm, systolic, diastolic, mean flow velocity; ONSD, optic nerve sheath diameter; ICP_TCD_, intracranial pressure measured with transcranial Doppler (TCD); CPP, cerebral perfusion pressure*.

### Correlation Between the Changes of Quantitative CT Variables, Respiratory Mechanics, and ICP

**Figure 4:**
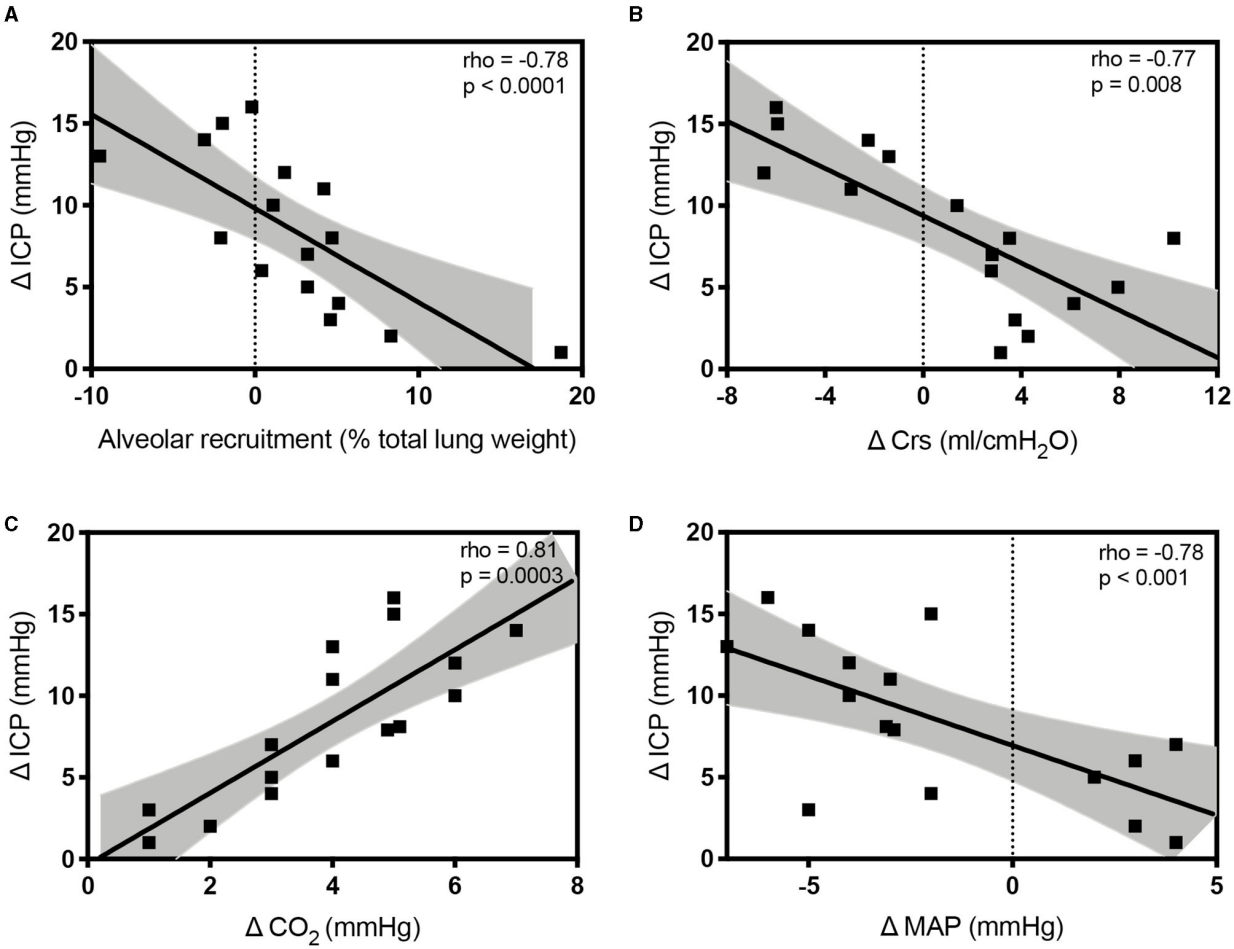
Scatterplots showing the linear association and correlation between alveolar recruitment **(A)**, Δ respiratory system compliance (Crs; **B**), Δ carbon dioxide (PaCO_2_; **C**), Δ mean arterial pressure (MAP; **D**) vs. Δ intracranial pressure (ICP) at different study time points. Dotted lines represent the 95% CIs for the linear regression.

The variations of lung volume and gas volume evaluated at qCT analysis were not correlated with the changes of ICP (rho = 0.05; *p* = 0.86 and rho = −0.07; *p* = 0.80, respectively). However, the amount of recruited tissue was inversely correlated with ΔICP (rho = −0.78; *p* = 0.0006). ΔCrs, ΔPlateau pressure, ΔPaCO_2_, and ΔMAP were significantly correlated with ΔICP (rho = −0.77; *p* = 0.008; rho = 0.54; *p* = 0.0002; rho = 0.81; *p* = 0.0003; rho = −0.64; *p* = 0.009, respectively; Figure 4).

At linear regression analysis, ΔPaCO_2_ (regression coefficient B = 0.96, 95% CI from 0.1 to 1.8, *p* = 0.028) and ΔCrs (B = −0.41, 95% CI from −0.647 to −0.183, *p* = 0.02) were the only independently variables associated with ΔICP. We did not observe correlations between the ICP increase and the following parameters assessed at PEEP 5 cmH_2_O: Crs, CO_2_, MAP, ONSD, ICP_TCD_, and invasive ICP (*p* > 0.40 in all correlations with ΔICP).

Correlation analysis between qCT variables, respiratory mechanics, and non-invasive ICP estimated through TCD and ONSD are presented in Supplementary Table 2. A significant correlation was found between the changes of ONSD and ICP (rho = 0.8096; *p* = 0.0003), but not between ICP_TCD_ and ICP (Supplementary Figure 1).

## Discussion

In a population of mechanically ventilated patients with acute brain injury, we found that (1) PEEP augmentation from 5 to 15 cmH_2_O may lead to higher oxygenation, PaCO_2_, and ICP values, with alveolar recruitment of 2.5% of total lung weight; (2) ICP increase with PEEP was correlated to higher PaCO_2_, poor alveolar recruitment, reduction of Crs, and decreased MAP; (3) baseline values of Crs, PaCO_2_, MAP, and ICP are not predictive for ICP increase with PEEP; and (4) changes in ONSD, but not ICP_TCD_, are correlated to changes in ICP.

To our knowledge, this is the first study quantitatively assessing alveolar recruitment and its distribution in the whole lung in mechanically ventilated patients with acute brain injury and its associations with changes in ICP and main physiological and clinical parameters.

Acute brain-injured patients with a clinical indication for chest CT, such as respiratory failure, and/or suspect of pneumonia were included in the study. This explains why the alveolar recruitment induced by PEEP was 2.5% of total lung weight, similar to a previous cohort of patients with COVID-19 (Ball et al., 2021). In fact, in the healthy population, the average lung weight is 930 g and gas volume 4,000 ml (Cressoni et al., 2013) in patients with ARDS-COVID-19, average lung weight is 1,500 g and gas volume 1,360 ml (Ball et al., 2021), whereas in our cohort of acute brain-injured patients average lung weight is 1,076 g and gas volume 1,693 ml.

Patients with acute brain injury admitted to ICU frequently require mechanical ventilation, and PEEP is often useful to support oxygenation, with the aim to ensure reliable oxygen delivery (Stevens et al., 2008; Borsellino et al., 2016; Del Sorbo et al., 2017) and minimize lung injury. However, mechanical ventilation and increased intrathoracic pressure can exert harmful effects on the brain due to complex physiological interactions between brain and lung compartments. The optimization of PEEP application in the general ICU population (Sutherasan et al., 2014; Algera et al., 2020) and in particular in patients with acute brain injury is still controversial. Recently, a systematic review of the literature revealed only marginal evidence for a specific ventilatory strategy in this group of patients (Robba et al., 2020), and only a few small physiologic studies have explored the effect of PEEP on intracranial dynamics (Caricato et al., 2005; Nemer et al., 2011). The use of higher PEEP may lead to possible negative hemodynamic effects, which could potentially lead to a reduction of MAP and therefore CPP. However, two small studies suggested that slow and progressive PEEP augmentation may be safe and can improve systemic and cerebral oxygenation without significant changes in ICP and CPP (Huynh et al., 2002; Nemer et al., 2011). Another important pathophysiological mechanism is the effect of respiratory mechanics on ICP. Caricato et al. (2005) demonstrated that in patients with low Crs (those with greater severity of lung injury and requiring higher PEEP), PEEP application had no important effects on cerebral and systemic hemodynamics. However, this finding was not confirmed in the present study. We observed a correlation between changes in ICP and worsening of Crs, but the absolute value of Crs at lower PEEP was not predictive for ICP increase with PEEP. We speculate that Crs measured at lower PEEP alone might not identify patients that will increase ICP at higher PEEP is not necessarily associated with the potential for lung recruitment. Patients with greater lung recruitment will improve lung gas distribution not resulting in ICP increase, while non-recruiters will over-inflate already aerated areas with a negative impact on dead space and possibly on venous return. In a prospective study that has 12 brain-injured patients, where 5 and 10 cmH_2_O of PEEP was randomly applied, patients defined as recruiters increased Crs and PaO_2_, while in non-recruiters Crs decreased and PaCO_2_ increased. Furthermore, ICP and jugular saturation remained constant in recruiters but significantly increased in non-recruiters, showing a significant correlation between changes in ICP, compliance, and PaCO_2_.

This suggests that PEEP may have a detrimental effect on ICP only when it causes alveolar hyperinflation leading to a significant increase in PaCO_2_, whereas when PEEP leads to good alveolar recruitment, ICP does not change.

Our results and previous evidence suggest that a precise evaluation of respiratory mechanics and gas exchange modifications may be of great importance in the assessment of recruitment. Comparative studies have shown that the only possible method to evaluate the amount of collapsed lung tissue regaining inflation is the CT scan (Gattinoni et al., 2017), thus making our study unique in the description of the pathophysiological effects of PEEP on the intracranial compartment, based on the characteristics of lung morphology. Our results show that in patients with acute brain injury increased PaCO_2_ and reduction of CPP and Crs with PEEP are the main factors associated with increased ICP. In fact, the potential mechanisms related to the overall ICP increase observed in our cohort might be related to the increase of PaCO_2_ and reduction of CPP. However, as MAP did not change, this latter mechanism was probably related to a reduction of intracranial compliance consequent to the supine position, thus possibly reducing jugular venous outflow.

We also demonstrated that the amount of alveolar recruitment is an important determinant of changes in ICP, thus suggesting that in patients with good response to alveolar recruitment, which leads to the improvement of Crs without affecting hemodynamic status and without causing alveolar hyperdistension of patients and therefore increased PaCO_2_, PEEP augmentation might be safe.

All in it, the principles for PEEP safety and titration in patients with acute brain injury seem not to be importantly different from those applied in the general ICU population and should take into account hemodynamic status, respiratory mechanics, and CT findings of patients (Ball et al., 2021). Indeed, a recent expert consensus (Robba et al., 2020) suggested that in brain-injured patients the levels of PEEP should be the same as for the general critically ill population. Similarly, a survey of the European Society of Intensive Care (Stocchetti et al., 2014) and a recent large multicenter study (Tejerina et al., 2021) suggest that moderate-high levels of PEEP are currently part of the clinical practice of neurocritical care physicians.

Finally, we explored the potential role of non-invasive ICP methods for the evaluation of changes of ICP after PEEP application. We found no significant correlation between non-invasive methods and qCT or respiratory mechanics data. This suggests that invasive ICP methods should be always considered as the gold standard for the evaluation of cerebral hemodynamics (Robba et al., 2015). However, changes in ONSD seem to be correlated with changes in ICP, thus making this tool a promising method for the bedside assessment of intracranial modifications when ICP is not available or contraindicated (Robba et al., 2018).

There are several limitations in our study that deserve to be mentioned. First, the sample size is small, despite similar to previous physiological studies exploring the effect of PEEP on lung recruitment (Mascia et al., 2005; Nemer et al., 2011; Mauri et al., 2016, 2020).

Second, in our center, a CT scan with double PEEP is routinely performed in selected patients with acute brain-injured patients, but only when CT is clinically indicated and in sufficiently stable patients. Therefore, patients were affected by brain damage of different nature and were heterogeneous as for comorbidities and lung damage.

Third, we cannot exclude that different ventilator setting may have led to different results (Gattinoni et al., 2006). However, we standardized mechanical ventilator settings, respiratory mechanics evaluation, and arterial blood gases measurement. In addition, we used a relatively short time for high PEEP exposure before repetition of CT. However, studies showed that most changes in volume and recruitment occur in this timeframe and that most respiratory units recruit below 30 cmH_2_O (Katz et al., 1981; Crotti et al., 2001). In fact, we were able to detect a clear recruitment effect in some patients. In addition, more data and details regarding hemodynamics and cardiac performance would have added greater insights regarding the effect of PEEP on cardiac function; however, unfortunately, we do not routinely perform in our institution echocardiography or carotid flow assessment during PEEP challenge. Finally, patients were in a supine position during CT, and this might have led to an increase of ICP regardless of the implementation of PEEP.

## Conclusions

Quantitative CT can help in the assessment of lung recruitability and the effect of different PEEP levels on ICP. The main factors associated with an increase of ICP after PEEP augmentation include worsening of Crs, reduction of MAP, low lung recruitment, and increased PaCO_2._ The potential benefits of PEEP augmentation in acute brain-injured patients should take into account hemodynamic status, respiratory mechanics, and lung morphology of patients. Further research is warranted to assess the effect of PEEP on ICP and the application of non-invasive ICP methods in this context.

## Data Availability Statement

The raw data supporting the conclusions of this article will be made available by the authors, without undue reservation.

## Ethics Statement

The studies involving human participants were reviewed and approved by Comitato Etico Genova, Liguria. The patients/participants provided their written informed consent to participate in this study.

## Author Contributions

CR, LB, PR, and PP designed the study and the methods. CR, SN, and DB collected the data. LB and CR performed the statistical analysis. All the authors participated in data evaluation, analysis, manuscript revision, and final manuscript approval.

## Conflict of Interest

The authors declare that the research was conducted in the absence of any commercial or financial relationships that could be construed as a potential conflict of interest.

## Publisher's Note

All claims expressed in this article are solely those of the authors and do not necessarily represent those of their affiliated organizations, or those of the publisher, the editors and the reviewers. Any product that may be evaluated in this article, or claim that may be made by its manufacturer, is not guaranteed or endorsed by the publisher.
