# Supplementary material for: Effects of Positive End-Expiratory Pressure on Lung Recruitment, Respiratory Mechanics, and Intracranial Pressure in Mechanically Ventilated Brain-Injured Patients
Source: Front Physiol. 2021 Oct 18;12:711273. doi: 10.3389/fphys.2021.711273 (PMC8558243; doi:10.3389/fphys.2021.711273)

Effect of positive end-expiratory pressure and lung recruitment on intracranial pressure in mechanically ventilated brain injured patients: a lung quantitative Computed Tomography study

Chiara Robba^1,2*^, Lorenzo Ball^1,2*^, Stefano Nogas^1^, Denise Battaglini^1^, Antonio Messina^3^, Iole Brunetti^1^, Giuseppe Minetti^4^, Lucio Castellan^4^, Patricia RM Rocco^5^and Paolo Pelosi^1,2^

^1^Anesthesia and Intensive Care, Ospedale Policlinico San Martino, IRCCS per l’Oncologia e le Neuroscienze, Genoa, Italy

^2^Department of Surgical Sciences and Integrated Diagnostics (DISC)

^3^ Humanitas Clinical and Research Center – IRCCS, Rozzano, MI, Italy

^4^Radiology Department Ospedale Policlinico San Martino, IRCCS per l’Oncologia e le Neuroscienze, Genoa, Italy

^5^Federal University of Rio de Janeiro, Rio de Janeiro, Brazil

**SUMMARY OF CONTENTS**

**Table S1.** “Strengthening the Reporting of Observational Studies in Epidemiology (STROBE)” statement guidelines……………….page 3

**File 1.** Clinical rationale for PEEP Test, Gas exchange and respiratory mechanics calculation……………………………………………………page 8

**Table S2.** Correlation between quantitative CT variables, respiratory system compliance and carbon dioxide values

with non invasive intracranial pressure…………………………………………………………………………………………………………………………………….page 12

**Figure S1.** Scatterplots showing the linear association and correlation between variations of invasive intracranial

pressure, and non in invasive ICP methods………………………………………………………………………………………………………………………………page 13

**Table S1.** “Strengthening the Reporting of Observational Studies in Epidemiology (STROBE)” statement guidelines.

|  | **Item No.** | **Recommendation** | **Page  No.** | **Relevant text from manuscript** | | |  |  |
| --- | --- | --- | --- | --- | --- | --- | --- | --- |
| **Title and abstract** | 1 | (*a*) Indicate the study’s design with a commonly used term in the title or the abstract | 1 |  | | | | |
|  |  | (*b*) Provide in the abstract an informative and balanced summary of what was done and what was found | 3 |  | | | |  |
| **Introduction** | | | |  | | |  |  |
| Background/rationale | 2 | Explain the scientific background and rationale for the investigation being reported | 5 |  |  |  |  |  |
| Objectives | 3 | State specific objectives, including any prespecified hypotheses | 5 |  |  |  |  |  |
| **Methods** | | | |  | | |  |  |
| Study design | 4 | Present key elements of study design early in the paper | 5 |  | |  |  |  |
| Setting | 5 | Describe the setting, locations, and relevant dates, including periods of recruitment, exposure, follow-up, and data collection | 5-6 |  | |  |  |  |
| Participants | 6 | (*a*) *Cohort study*—Give the eligibility criteria, and the sources and methods of selection of participants. Describe methods of follow-up | 5-6 |  | |  |  |  |
|  |  |  |  |  | |  |  |  |
| Variables | 7 | Clearly define all outcomes, exposures, predictors, potential confounders, and effect modifiers. Give diagnostic criteria, if applicable | 6 |  | |  |  |  |
| Data sources/ measurement | 8* | For each variable of interest, give sources of data and details of methods of assessment (measurement). Describe comparability of assessment methods if there is more than one group | *6-ESM* |  | | |  |  |
| Bias | 9 | Describe any efforts to address potential sources of bias | ESM |  | | |  |  |
| Study size | 10 | Explain how the study size was arrived at | ESM |  | | |  |  |

Continued on next page

| Quantitative variables | 11 | Explain how quantitative variables were handled in the analyses. If applicable, describe which groupings were chosen and why | ESM |  | | | |  |  |  |
| --- | --- | --- | --- | --- | --- | --- | --- | --- | --- | --- |
| Statistical methods | 12 | (*a*) Describe all statistical methods, including those used to control for confounding | 6,ESM |  | | | |  |  |  |
|  |  | (*b*) Describe any methods used to examine subgroups and interactions |  | |  | | | |  |  |
|  |  | (*c*) Explain how missing data were addressed |  | |  | | | |  |  |
|  |  | ( |  | | |  | | | |  |
|  |  | (*e*) Describe any sensitivity analyses |  | | |  | | | |  |
| **Results** | | | | | | | | | | |
| Participants | 13* | (a) Report numbers of individuals at each stage of study—eg numbers potentially eligible, examined for eligibility, confirmed eligible, included in the study, completing follow-up, and analysed |  | | | |  | | | |
|  |  | (b) Give reasons for non-participation at each stage |  | | | |  | | | |
|  |  | (c) Consider use of a flow diagram |  | | | |  | | | |
| Descriptive data | 14* | (a) Give characteristics of study participants (eg demographic, clinical, social) and information on exposures and potential confounders | 6,7 | | | |  | | | |
|  |  | (b) Indicate number of participants with missing data for each variable of interest |  | | | |  | | | |
|  |  | (c) *Cohort study*—Summarise follow-up time (eg, average and total amount) |  | | | |  | | | |
| Outcome data | 15* | *Cohort study*—Report numbers of outcome events or summary measures over time | 6-9 | | | |  | | | |
|  |  | *Case-control study—*Report numbers in each exposure category, or summary measures of exposure |  | | | |  | | | |
|  |  | *Cross-sectional study—*Report numbers of outcome events or summary measures |  | | | |  | | | |
| Main results | 16 | (*a*) Give unadjusted estimates and, if applicable, confounder-adjusted estimates and their precision (eg, 95% confidence interval). Make clear which confounders were adjusted for and why they were included | 6-9 | | | |  | | | |
|  |  | (*b*) Report category boundaries when continuous variables were categorized |  | | | |  | | | |
|  |  | (*c*) If relevant, consider translating estimates of relative risk into absolute risk for a meaningful time period |  | | | |  | | | |

Continued on next page

| Other analyses | 17 | Report other analyses done—eg analyses of subgroups and interactions, and sensitivity analyses | ESM |  |  |
| --- | --- | --- | --- | --- | --- |
| **Discussion** | | | | |  |
| Key results | 18 | Summarise key results with reference to study objectives | 9 |  |  |
| Limitations | 19 | Discuss limitations of the study, taking into account sources of potential bias or imprecision. Discuss both direction and magnitude of any potential bias | 11 |  | |
| Interpretation | 20 | Give a cautious overall interpretation of results considering objectives, limitations, multiplicity of analyses, results from similar studies, and other relevant evidence | 10-11 |  | |
| Generalisability | 21 | Discuss the generalisability (external validity) of the study results | 10-11 |  | |
| **Other information** | |  | | |  |
| Funding | 22 | Give the source of funding and the role of the funders for the present study and, if applicable, for the original study on which the present article is based | 16 |  |  |

*Give information separately for cases and controls in case-control studies and, if applicable, for exposed and unexposed groups in cohort and cross-sectional studies.

**Note:** An Explanation and Elaboration article discusses each checklist item and gives methodological background and published examples of transparent reporting. The STROBE checklist is best used in conjunction with this article (freely available on the Web sites of PLoS Medicine at http://www.plosmedicine.org/, Annals of Internal Medicine at http://www.annals.org/, and Epidemiology at http://www.epidem.com/). Information on the STROBE Initiative is available at www.strobe-statement.org

**Gas exchange and respiratory mechanics**

Blood gas analyses and ventilation parameters were collected in all patients on the day of the CT scan. The ventilatory ratio was computed as:

| *Ventilatory Ratio= Minute ventilation(ml/min)×PaCO_2_(mmHg)*  *Predicted body weight (kg)×100×37.5* |
| --- |

The ventilatory ratio is an estimate of ventilation impairment and is known to correlate with physiologic dead-space fraction in COVID-19 patients^1^.

We assessed gas exchange and respiratory mechanics at PEEP 5 and 15 cmH2O. Shunt was calculated using the FiO_2_ from of 1.0 to estimate venous admixture and at a FiO_2_ of 0.5, the latter value being arbitrarily chosen to explore the effects of FiO_2_ changes on oxygenation. All four possible PEEP/FiO_2_ combinations were tested. Blood gas analyses were performed after allowing 5 minutes for stabilization, and respiratory mechanics were assessed within 2 h from the CT scan and included estimation of venous admixture based on arterial and central venous blood gas samples.

Venous admixture was assessed at FiO_2_ 1.0 at the two PEEP levels with the following formula:

$$\frac{\dot{Q}_{VA}}{\dot{Q}_{T}}=\frac{CcO_{2}-CaO_{2}}{CcO_{2}-CvO_{2}}$$

Where the capillary oxygen content (CcO_2_) was computed as:

$${CcO}_{2}=\left( {P_{A}O}_{2}\cdot0.0031 \right)+\left[ Hb \right]\cdot100\%\cdot1.36$$

assuming

$${P_{A}O}_{2}=\left( 760 mmHg-47 mmHg \right)\cdot\left( 1-\frac{{PaCO}_{2}}{0.8} \right)$$

The arterial oxygen content (CaO_2_) was computed as:

$${CaO}_{2}=\left( {PaO}_{2}\cdot0.0031 \right)+\left[ Hb \right]\cdot{SaO}_{2}\cdot1.36$$

The venous oxygen content (CvO_2_) was estimated from a central venous blood sample as:

$${CvO}_{2}=\left( {PvO}_{2}\cdot0.0031 \right)+\left[ Hb \right]\cdot{SvO}_{2}\cdot1.36$$

The compliance of the respiratory system was computed as:

$$C_{rs}=\frac{V_{T}}{P_{plat}-PEEP}$$

Where V_T_ is the tidal volume and P_plat_ the plateau pressure measured after a manual inspiratory hold.

**Two-PEEP CT acquisition and analysis**

Alveolar recruitment was defined as the percent of lung weight accounted for by non-aerated tissue in which aeration was restored increasing PEEP from 5 to 15 cmH_2_O, *i.e.*:

$$Recruitment= \left( \frac{{Nonaerated lung tissue}_{PEEP 8cmH_{2}O}}{{Total lung weight}_{PEEP 8cmH_{2}O}}-\frac{{Nonaerated lung tissue}_{PEEP 16 cmH_{2}O}}{{Total lung weight}_{PEEP 16 cmH_{2}O}} \right)\times100$$

The lung excess lung weight was calculated as percent difference of the CT-measured lung weight at 5 cmH_2_O PEEP compared to the expected CT lung weight of a supine healthy patient, as follows:

$Excess lung weight \left( \% \right)= \frac{{Lung weight}_{measured, PEEP 8cmH_{2}O}-{Lung weight}_{expected}}{{Lung weight}_{expected}}\times100$,

where

$${Lung weight}_{expected}\left( g \right)=-1806.1+1633.7\times height$$

References

1. Diehl JL, Peron N, Chocron R, et al. Respiratory mechanics and gas exchanges in the early course of COVID-19 ARDS: a hypothesis-generating study. Ann Intensive Care 2020;

| **Parameter** | **Delta ICP_TCD_** | | **Delta ONSD** | |
| --- | --- | --- | --- | --- |
|  | **r** | **p value** | **r** | **p value** |
| Delta Total lung volume (ml) | -0.9346 | <0.0001 | -0.0816 | 0.7628 |
| Delta Gas volume (ml) | -0.9388 | <0.001 | -0.0786 | 0.7713 |
| Delta Recruitment | 0.04096 | 0.8799 | 0.2428 | 0.3612 |
| Delta Crs | -0.2188 | 0.4156 | -0.2804 | 0.2903 |
| Delta PaCO_2_ | 0.3671 | 0.1612 | -0.014 | 0.9580 |

**Table S2.** Correlation between the variations of total lung volume, gas volume, recruitment evaluated through quantitative CT analysis, respiratory system compliance (Crs) and carbon dioxide (PaCO_2_) values with non invasive intracranial pressure methods at 5 and 15 cmH_2_0 of PEEP. PEEP; positive end expiratory pressure, ONSD; optic nerve sheath diameter, ICP_TCD_; non invasive intracranial pressure measured using Transcranial Doppler.

**Figure S1.** Scatterplots showing the linear association and correlation between variations of invasive intracranial pressure (Delta ICP), and non in invasive ICP methods between 5 and 15 cmH_2_0 of PEEP. Dotted lines represent that 95% confidence intervals for the linear regression.

PEEP; positive end expiratory pressure, ONSD; optic nerve sheath diameter, ICP_TCD_; non invasive intracranial pressure measured using Transcranial Doppler.


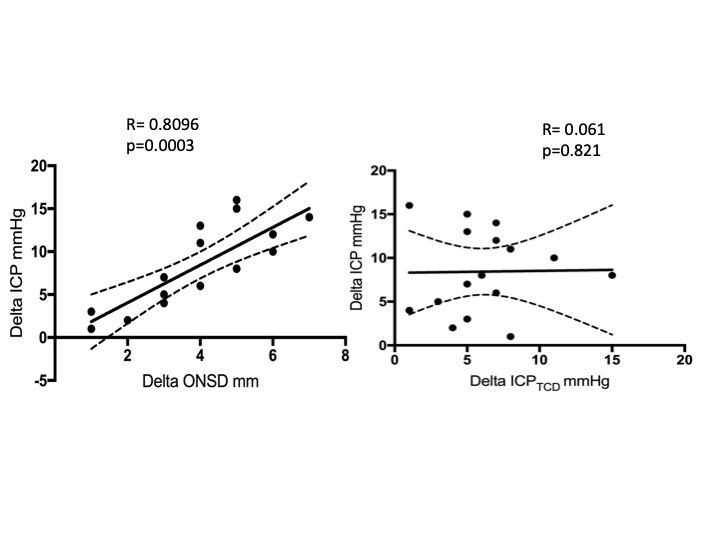

Supplement: Supplementary file 1 [file Data_Sheet_1.docx]
